# Mouse lung contains endothelial progenitors with high capacity to form blood and lymphatic vessels

**DOI:** 10.1186/1471-2121-11-50

**Published:** 2010-07-01

**Authors:** Judith Schniedermann, Moritz Rennecke, Kerstin Buttler, Georg Richter, Anna-Maria Städtler, Susanne Norgall, Muhammad Badar, Bernhard Barleon, Tobias May, Jörg Wilting, Herbert A Weich

**Affiliations:** 1 Helmholtz Centre for Infection Research, Division Molecular Biotechnology, Department of Gene Regulation, Braunschweig, Germany; 2 Department of Anatomy and Cell Biology, Georg-August-University, Goettingen, Germany; 3 RELIATech Research Lab, Receptor Ligand Technologies GmbH, Wolfenbuettel, Germany

## Abstract

**Background:**

Postnatal endothelial progenitor cells (EPCs) have been successfully isolated from whole bone marrow, blood and the walls of conduit vessels. They can, therefore, be classified into circulating and resident progenitor cells. The differentiation capacity of resident lung endothelial progenitor cells from mouse has not been evaluated.

**Results:**

In an attempt to isolate differentiated mature endothelial cells from mouse lung we found that the lung contains EPCs with a high vasculogenic capacity and capability of *de novo *vasculogenesis for blood and lymph vessels.

Mouse lung microvascular endothelial cells (MLMVECs) were isolated by selection of CD31^+ ^cells. Whereas the majority of the CD31^+ ^cells did not divide, some scattered cells started to proliferate giving rise to large colonies (> 3000 cells/colony). These highly dividing cells possess the capacity to integrate into various types of vessels including blood and lymph vessels unveiling the existence of local microvascular endothelial progenitor cells (LMEPCs) in adult mouse lung. EPCs could be amplified > passage 30 and still expressed panendothelial markers as well as the progenitor cell antigens, but not antigens for immune cells and hematopoietic stem cells. A high percentage of these cells are also positive for Lyve1, Prox1, podoplanin and VEGFR-3 indicating that a considerabe fraction of the cells are committed to develop lymphatic endothelium. Clonogenic highly proliferating cells from limiting dilution assays were also bipotent. Combined *in vitro *and *in vivo *spheroid and matrigel assays revealed that these EPCs exhibit vasculogenic capacity by forming functional blood and lymph vessels.

**Conclusion:**

The lung contains large numbers of EPCs that display commitment for both types of vessels, suggesting that lung blood and lymphatic endothelial cells are derived from a single progenitor cell.

## Background

In the developing embryo blood vessels and later also lymphatic vessels are formed via an initial process of vasculogenesis. This is followed by sprouting and intussusceptive growth of the vessels, termed angiogenesis for blood vessels and lymphangiogenesis for lymph vessels. These mechanisms give rise to a complete blood and lymphvascular system consisting of arteries, veins, capillaries and collectors. Endothelial cells (ECs) are specified according to the circulatory system (blood *versus *lymph) and to the vessel type (vein, artery, capillary) to which they belong [1]. However, ECs from diseased tissues can have different molecular markers and characteristics from those found in normal vascular beds [2,3]. The interface formed by ECs between blood or lymph and the surrounding tissue has different physiological functions in different organs and is an important attribute for tissue homeostasis. Although differentiated endothelium hardly proliferates in most organs, under pathological conditions and in female reproductive organs ECs are replaced and proliferate to support tissue growth and to preserve vascular and organ homeostasis [4,5]. Already in the 1970s, the presence of fast-growing endothelial cells within niches of the vessel intima was postulated [6]. These early findings together with more recent data suggest that the turnover rate of ECs in conduit blood vessels *in vivo *is in the range of several years [4,7]. Heterogeneity of endothelial cells does not only exist with regard to blood and lymphatic vessels, but also along the arterial-capillary-venule axis, and between capillaries of specific tissues and organs [4,5]. Physiological capillary growth can achieve high rates, for example, cyclically capillary growth is found in the corpus luteum to transport blood to the granulosa cells during the menstrual cycle [8]. Thereby, the proliferation rate of ECs is comparable in its extent to fast growing tumours [9]. Therefore, at least some microvascular ECs and ECs in specific niches possess high endogenous proliferation capacities *in vivo*, as well as high angiogenic potential as part of their physiological role.

Under *in vitro *conditions, both microvascular and macrovascular ECs can reestablish their proliferative phenotype but it has been shown that, for example, pulmonary microvascular ECs from rats grow approximately twice as fast as pulmonary artery ECs [10,11]. At the molecular level, microvascular ECs possess higher expression of cell cycle regulating genes and inactivation of antimitogenic proteins [12]. Based on these results, it is not clear whether all microvascular ECs exhibit a higher proliferation rate or whether only a subpopulation of replication-competent cells, as suggested by Schwarz and Benditt [6] from their *in vivo *findings, account for such high proliferation rates.

More recently it has been demonstrated that preparations of human large vessel endothelial cells contain a moderate number of cells that have a higher single-cell-layer growth potential and show endothelial colony-forming activity [13]. Thereby, it has been demonstrated that ~50% of primary cells seeded as single cells grow, but only ~12.5% of these cells displayed a high proliferative behaviour. Based on their high capacity for self-renewal and regeneration, fast proliferating endothelial colony-forming cells were considered to be endothelial progenitors (EPCs) [13-15]. Interestingly, these isolated EPCs demonstrated an intrinsic vasculogenic capacity, as shown by their capability to form *de novo *vessels *in vivo*, the most striking function for the endothelium. Recently, similar cells were found in rats where they were isolated from the lung as resident EPCs [11]. A comprehensive list of the different EPCs, their nomenclature, source and surface markers can be found in a very recently published review [16]. These results support earlier observations by Schwartz and Benditt for the endothelium of the vessel wall and prove that EC populations from conduit vessels contain progenitor niches [6].

In recent years, we have established numerous endothelial cell lines from different mouse strains and immortalized them by transduction with polyoma middle T (PmT) virus [17]. However, immortalized ECs may lose some of their typical characteristics, and we, therefore, looked for a reliable source of replication-competent mouse ECs. We finally used lung tissue for the isolation of mouse microvascular endothelial cells and discovered that lung tissue contains a large number of fast-growing resident EPCs, with vasculogenic capacity *in vitro *and *in vivo*, and capable of forming both blood and lymphatic capillaries. This is the first report, which suggests that blood and lymphatic endothelial cells in the lung are derived from a common progenitor cell.

## Results

### Mouse lungs contain ECs with high proliferation capacity

**Figure 1:**
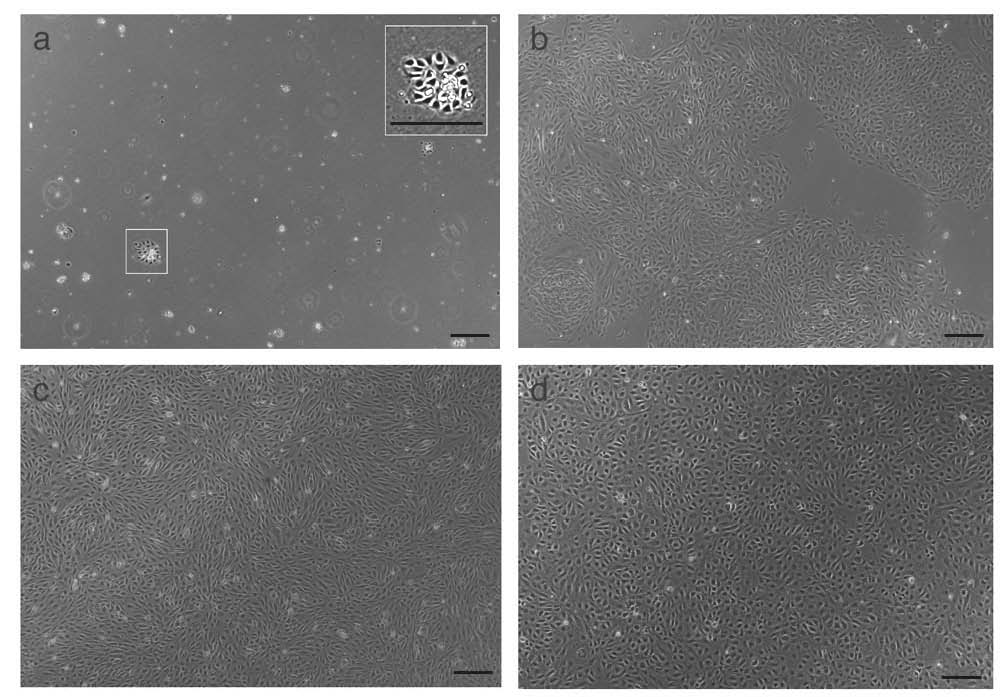
**Cultured mouse lung EPCs at different time points after isolation**. Three lungs were used for magneto bead isolation and cells were plated in one well of a 12- or 6-well plate. a) One day after cell isolation, the inset in a) shows a small colony with high replication capacity. b) Homogeneous large colonies with high proliferation capacity have formed six days after isolation. c) Lung EPCs in passage 12 and d) in passage 31. Scale bar = 200 μm.

Using adult mouse lung for magneto-bead selection of CD31^+ ^endothelial cells, we discovered that the lung contains endothelial cells, which have a high proliferative capacity. When lungs from three animals were combined and used for tissue homogenisation followed by anti-CD31 cell sorting, only very few isolated cells started to proliferate (Figure 1a). Six days after cell isolation, individual colonies (~20-50 from one preparation) revealed high-proliferative potential. These cells were capable of forming colonies of > 4,000 cells (Figure 1b). After confluence, cells could be subcultured by splitting them 1:5 - 1:20 and cells proliferated at a high rate. In early passages, contaminating cells were removed when necessary by a second CD31 FACS sorting. Cells were stable during cell culture conditions up to passage 30-40 (Figure 1c, d).

**Figure 2:**
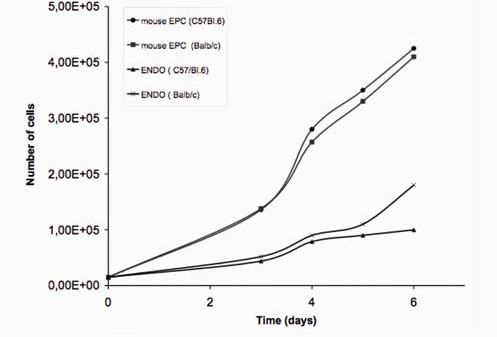
**Mouse lung EPCs show a high proliferation capacity if compared with mouse endothelioma cells derived from the same mouse strain**. Serum-stimulated growth was compared in equivalently seeded EPCs and endothelioma cells over a 6-day period. After a lag phase, a significantly increased growth rate appears in EPCs compared to endothelioma cells.

Proliferation of mouse lung EPCs was compared with endothelioma cells isolated from a cognate mouse strain over a six-day period. After a two-day lag phase, the number of EPCs was 3.2-fold higher than that of endothelioma cells on day 4. The significant difference in cell growth was also present after 6 days (Figure 2). The data show that normal mouse lung EPCs grow significantly faster than PmT oncogene-transformed mouse endothelioma cells. Proliferations experiments in the presence of VEGF-A show, that the growth factor has only a low effect (~20% at 20 ng/ml VEGF-A) to enhance proliferation in vitro (data not shown). Although the mechanism for such a high proliferation rate remains unknown, the data underline the existence of highly regenerative endothelial precursor cells in the murine lung.

A key feature of stem cells is their ability to maintain stem cell properties after division, known as self-renewal. To exclude the possibility of overestimation due to cell aggregation or formation of secondary colonies, a single-cell clonogenic assay was performed by culturing single cells by limiting dilution in individual wells of 96-well plates. We found that ~15% (± 3.6%) of the wells contained highly proliferating colonies. These wells were 80-100% confluent after 18-20 days and had a cell number of 4.3-10^4 ^(± 1.4 × 10^4^) cells/well at the end of the experiment (day 28). From the highly proliferating clonal MLMVECs six lines were established and maintained in culture for several passages. Clones were cryopreserved at passage 15 and 20. From these cultures four clones were further characterized for BEC (blood endothelial cell) and LEC (lymph endothelial cell) markers (see below). The abundance of endothelial precursor cells within the MLMVEC population provides an explanation for the accelerated growth of MLMVECs as compared with mouse endothelioma cells.

### Mouse lung primary EPCs in early culture are heterogeneous and have the potential to differentiate into lymphatic and blood endothelial cells

**Figure 3:**
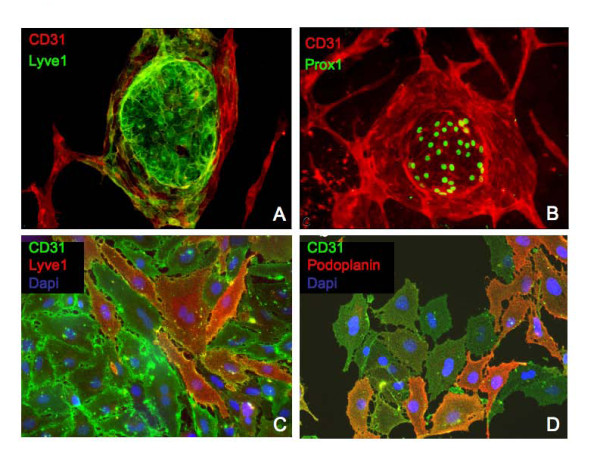
**Heterogeneity of mouse EPCs immediately after isolation and EPC subclones are bipotent**. A-B) CD31^+ ^BECs surround LECs, which are in the centre of a growing colony and which are Lyve1 positive. BECs are more positive for CD31 than LECs and all LECs specifically express the transcription factor Prox1. C-D) After a single-cell clonogenic assay all cells from two representative subclones (D5 and B3) are positive for CD31, a panendothelial marker. Only cells differentiated into lymph endothelial cells (LECs) are Lyve1 or podoplanin positive. 150-200-fold.

In one of the earliest reports on the isolation of human BECs and LECs from a dermal cell suspension, it has been shown that the cells form separate homotypic clusters [18]. Early after CD31-mediated isolation, our cells formed colonies as described previously for human ECs, in which a cluster of LECs is surrounded by BECs (Figure 3; additional file 1). The LECs are positive for the three key lymphendothelial markers Podoplanin, Lyve1 and Prox1. BECs, which are more strongly positive for CD31, are negative for Lyve and Prox1. Occasionally, BECs generate sprout-like structures in these early cultures (Figure 3). Some cells express low levels of Lyve1 as indicated by the yellow color. After Lyve1 cell sorting clusters were predominantly made of LECs (additional file 1). These observations were made during early passages and we speculated that at least some of the isolated progenitor cells have a dual capacity to differentiate into BECs and LECs *in vitro*.

To study the hypothesis if a dual differentiation capacity can be found in endothelial precursor cells, a single-cell clonogenic assay was performed. Four subclones derived from a single-cell suspension were used to study the expression of CD31, Lyve1, Podoplanin and Prox1. The results showed very clearly, that both endothelial cell types could be found in each subclone, indicating that the precursor cells are bipotent. A representative result is shown in Figure 3. Single EPCs from microvascular lung have the ability to generate lymph and blood vessel endothelial cells under *in vitro *conditions.

We also observed that isolated mouse lung endothelial cells were either Lyve1^+ ^or Lyve1^- ^and that the majority seems to represent LECs and the minority BECs (additional file 2). Upon Lyve1 and CD31 cell sorting and further culturing for 10 days, a high proportion of the CD31^+^/Lyve1^- ^cells were either positive (~71%) or still negative for Lyve1 (additional file 3), - adjusting to a similar relationship as before (additional file 2). This was again visible after another passage of these cells (~78% Lyve1^+ ^cells). This may indicate that early isolated EPCs may have the potential to differentiate into BECs and LECs. Due to the high proliferative capacity of the precursor cells, differentiated cells are "diluted" during cultivation and a fixed segregation of endothelial cell types may occur.

### Mouse lung EPCs are capable of vascular sprout formation

**Figure 4:**
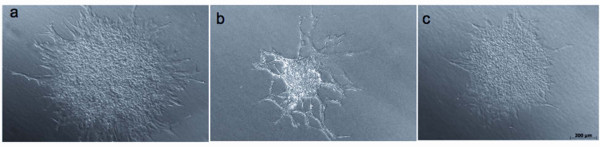
**Mouse EPCs from lung exhibit rapid *in vitro *tube formation in Matrigel**. a) Low passage EPCs were examined after seeding on Matrigel-coated plates after a 5-day period. b) Same as in a) but with high passage EPCs after a 14-day period. c) Mouse endothelioma cells were examined after seeding on Matrigel-coated plates after a 5-day period.

One of the key characteristics of endothelial cells is the intrinsic capability to form vascular networks on Matrigel *in vitro*. We, therefore, examined the capability of our lung EPCs at low passage and at high passage and of immortalized endothelioma cells to form networks on Matrigel-coated wells. To increase differentiation into radial sprout-like structures, cells were predifferentiated overnight as spheroids in hanging drops. Mouse EPCs generate capillary sprouts as early as 3-5 days after seeding (Figure 4a). Longer exposure of the cells generates extensive network formation and more cells from the spheroid are involved in vascular sprout formation (Figure 4b). However, immortalized endotheliomas are also able to differentiate into vascular sprouts (Figure 4c). The length of these structures was measured 5 days after plating and resulted in an average range of 200-250 μm. The growth factor combination of hepatocyte growth factor (HGF) and endothelial cell growth factor (ECGF or endothelial cell growth supplement) had the most prominent effect on sprout length (additional file 4). The data indicate that mouse EPCs from the lung have the ability to form vascular networks under *in vitro *conditions.

### Characterization of mouse lung EPC and endothelial markers

**Figure 5:**
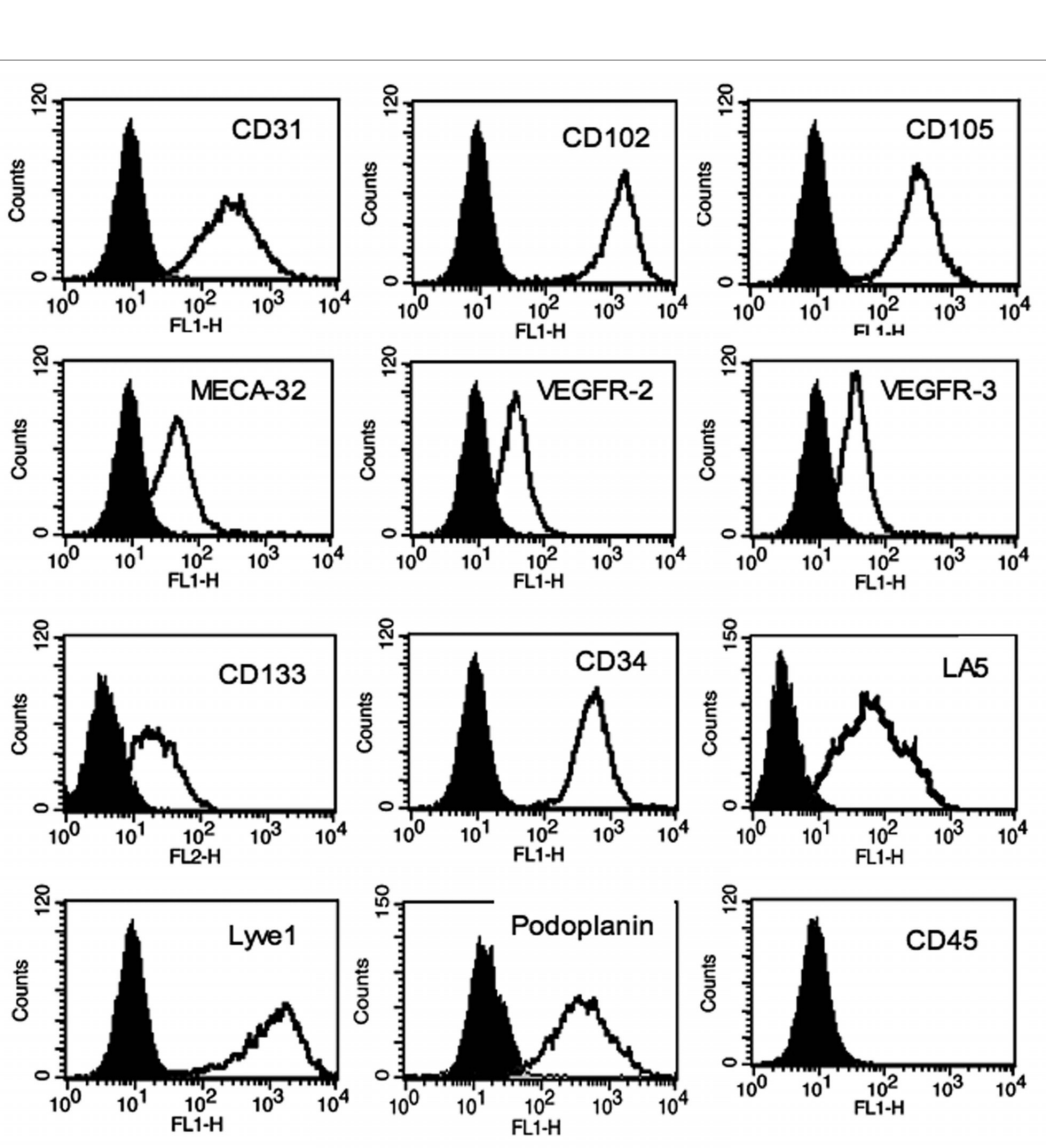
**Mouse lung EPCs retain a microvascular endothelial phenotype**. Immunophenotyping of cells was performed with flow cytometric analyses. Mouse lung EPCs express the pan-endothelial markers CD31, CD102 and CD105. They also express circulating EPC markers CD34, CD133, vascular endothelial growth factor receptors 2 and 3 (VEGFR-2 + VEGFR-3). The new marker LA5 and MECA32/PV1 for blood endothelial cells (BEC) is also expressed as well as the lymphatic markers Lyve1 and podoplanin. The pan-lymphocyte marker CD45 was not detectable.

The use of cell surface markers for the characterization of cell type specificity has greatly improved the phenotyping of mouse endothelial cells, because these change their morphology depending on passage number and rate of confluence. In this study, we sought to determine whether mouse EPCs express panendothelial markers as well as markers for progenitor cells and for BECs and LECs. The key endothelial marker, besides cobblestone morphology, is CD31 (PECAM-1). Only cells from colonies were used in this study, which were more than 90% positive for this marker. Some cells from early passages were CD31 FACS sorted again if necessary. Routine FACS analyses between passage 4-8 indicated that more than 99% (99,5 ± 0.2%) of MLMVECs were CD31^+^. Other panendothelial markers include CD102 (ICAM-2) and CD105 (endoglin), which are also expressed in EPCs (Figure 5). MLMVECs at low passage were positive for CD144 (~92%) and the four subclones derived from a single cells suspension were 54-98% positive for CD144 (not shown). For *Griffonia simplicifolia *lectin, it was shown that this interacts with murine microvascular endothelial cells [11], which could also be confirmed in our study (data not shown). Thus, the isolated mouse lung EPCs express classical endothelial cell markers and display a microvascular phenotype. Further, we sought to determine whether the EPCs express markers for circulating and resident EPCs as originally shown by Asahara and colleagues for circulating cells and for resident rat EPC by Avarez and colleagues [11,19]. Expression of CD34 (mucosialin), VEGFR-2 (KDR), VEGFR-3 (Flt-4), and CD133 (prominin-1) are widely accepted markers for circulating EPC. Expression of CD34, VEGFR-2 and CD45 (leukocyte common antigen), but not CD133 define a population of resident conduit vessel wall EPCs [11]. Very similar to circulating and vessel wall-derived EPCs, mouse lung EPCs express CD34, CD133 and VEGFR-2 (Figure 5). However, cells were negative for CD45, for CD117 (c-kit or SCF receptor) and CD41, which are markers for leukocytes and hematopoietic stem cells (data not shown). As discussed before, our progenitor cells were also analyzed for specific LEC markers. Expression of Lyve1, VEGFR-3 and podoplanin indicated that a least a subpopulation of the cells are committed to lymphatic endothelial differentiation (Figure 5). On the other hand, these cells express LA5 and MECA-32/PV-1, markers very recently described for mouse blood endothelial cells [20]. From two isolates we have also made a two colour labeling using Lyve1 and MECA32/PV-1 or Lyve1/LA5 (additional file 5).

### Mouse EPCs are not transformed

**Figure 6:**
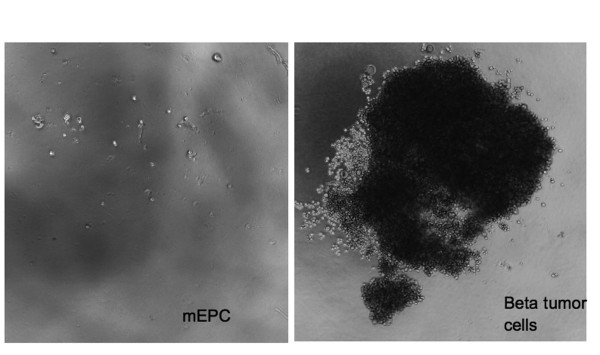
**Mouse EPCs do not display evidence of transformation**. The evaluation of anchorage-independent growth was examined in mouse EPCs and cells derived from a beta cell tumour seeded onto a soft agar matrix. Whereas EPCs did not grow on soft agar, beta tumour cells (used as control) formed frequent colonies during the 8 days of cultivation. 50-fold.

Based on the high proliferation rate and the high vasculogenic capacity under culture conditions, we investigated the possibility that these cells might be transformed and display a malignant phenotype. Therefore, we determined anchorage-independent cell growth in a 0.8% agar matrix. As a control, a mouse beta tumor cell line (βTC) was used in parallel with the same number of cells per well. Mouse EPCs did not display proliferative capacity, and no clusters of cells were found throughout the agar (Figure 6). Also, when EPCs from different isolations and passage numbers were used, we could not detect cluster formation or growth on soft agar during a period of 8 days. Beta cell tumors formed numerous large colonies of more than 1000 cells (Figure 6). These results support the observation that, although mouse lung EPCs are fast-growing cells with a capacity for self-renewal, they do not display any signs of malignancy or transformation.

### In vivo vessel formation of mouse EPCs in Matrigel implants

**Figure 7:**
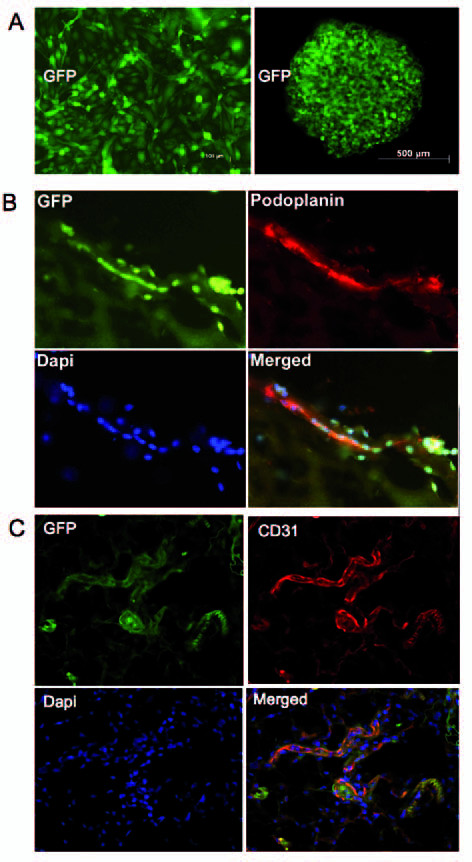
**In vivo studies of mouse lung EPCs after lentiviral transduction with GFP marker gene**. A) Cells as a monolayer (left) or as spheroids (right) used for Matrigel implantation into mice. B) *De novo *formation of lymphatic vessels as revealed by double staining with anti-GFP and anti-podoplanin (which is LEC-specific) in combination with nuclear Dapi staining. Note that the diagonal vessel in the picture expresses both markers. C) *De novo *formation of blood vessels as revealed by double staining with anti-GFP and anti-CD31 (strong CD31 staining is characteristic for BECs) in combination with nuclear Dapi staining. Erythrocytes in the vessels display green/yellow background fluorescence.

One of the key questions to be answered was whether mouse EPCs possess the ability to generate capillaries and larger blood and lymphatic vessels *in vivo*. This is a critical issue and should be displayed by any cell type denoted EPC. Before implantation, we marked cells by lentiviral transduction in order to express GFP, which allows for discrimination between endogenous and exogenous mouse endothelial cells. To facilitate differentiation, EPCs were cultured overnight as hanging drops, which allows cells to adhere to each other and to stop proliferation (Figure 7A). Mouse EPCs were then mixed in Matrigel (~ 400,000 cells/plug) and injected subcutaneously into the neck of a normal mouse. Plugs were harvested 10-11 days after injection. At that time, numerous blood vessels were found in all plugs that had been seeded with EPCs, whereas in control plugs without cells but with VEGF and bFGF, only a small number of blood vessels was present. Implanted EPCs could be visualized by staining with anti-GFP antibodies and we observed that cells started to line up into tube-like structures. Tubes which stained positive for podoplanin, a reliable marker for lymphatic vessels could be identified (Figure 7B). Additionally, cells positive for GFP and LA102, a marker for lymph endothelial cells, but negative for CD45 could be found in tube-like structures (additional file 7). Single cells, positive for GFP and DAPI but not involved in tube formation, were either podoplanin-positive or negative (Figure 7B). Additionally, GFP-transfected EPCs were also found in blood capillaries. Other Matrigel infiltrating cells, which were obviously immune cells and fibroblasts did not contribute to tube formation, and were GFP-negative. Triple staining with anti-GFP, anti-podoplanin and anti-CD31 indicated that blood and lymphatic networks could exist independently in the same implanted Matrigel plug (additional file 8). A dense network of numerous, perfused vessels could be seen, positive for CD31 and containing a chimeric pattern of GFP-positive and negative vessels (Figure 7C). These vessels obviously contained both endogenous ECs and implanted EPCs. For *de novo *vessel formation, we used EPCs from different isolations and found that all cells were able to generate vessels that were structurally similar to the vessels described above. The results illustrated that lung endothelial progenitor cells are able to generated functionally and structurally normal lymphatic and blood vessels.

## Discussion

### Endothelial precursor cells are found in different niches

Bone marrow as well as peripheral blood of adults and umbilical cord blood of newborns contain special sub-types of progenitor cells, which are able to differentiate into mature endothelial cells. Under conditions of tissue regeneration or hypoxia they contribute to re-endothelialization of denuded vessels and neo-angiogenesis [19,21]. These angiogenic cells have properties of angioblasts and are termed endothelial progenitor cells (EPCs). The three main cell surface markers of this cell type are CD133, CD34 and VEGFR-2/KDR. Within the systemic circulation, EPCs gradually lose their progenitor properties and start to express markers for mature endothelial cells, such as VE-cadherin and eNO synthase [13]. Cultured endothelial cells isolated from human umbilical vein or aorta contain a small amount of EPCs, which can be discriminated from mature, differentiated ECs by their proliferative, colony-forming potential [13]. Recently, human EPCs have also been characterized and isolated from the internal thoracic artery by their potential to form capillary sprouts. These macrovessel-wall derived EPCs express markers for mature endothelial cells after tumorigenic activation [22]. Very recently these precursors have been described as endothelial outgrowth cells (EOCs) and differentiated from EC-like cells and circulating endothelial cells [16]. Together, the data have led to the concept that EPCs are a heterogeneous group of precursor cells, which can be found in different niches, for instance bone marrow, circulating blood and vascular wall, participating in both new blood vessel formation (vasculogenesis) and vascular homeostasis [6,23].

In the present study we sought to determine whether adult mouse lungs can be used as donor organs for the isolation of vascular endothelial cells, to be used *for in vitro *and *in vivo *proliferation studies. We found microvascular ECs, which possess an intrinsic ability to proliferate at high rates and differentiate *in vitro *into lymph and blood vascular endothelial cells. Subpopulations of these cells segregate in early passages and form homogeneous clusters of BECs and LECs. A similar behaviour has been reported for cultured human dermal microvascular endothelial cells [18]. *In vivo*, predifferentiated microvascular endothelial cells from the mouse lung can undergo *de novo *hemangiogenesis and lymphangiogenesis. The high proliferative capacity of a subpopulation of cells and the heterogeneity of early isolated cells suggests the presence of progenitor cells as an essential part of lung vascular cells. Our findings demonstrated that in contrast to circulating or resident conduit vessel wall-derived EPCs, lung EPCs express classical endothelial markers as well as markers uniquely found in circulating progenitor cells. They also express markers typical for lymph endothelial cells and blood endothelial cells but lack the expression of leukocyte antigens as well as (hematopoietic) stem cell markers.

During a literature search for similar cells, we found a very recent report by Alvarez and colleagues, who describe that rat lung microvessels are enriched with endothelial progenitor cells (EPCs) [11]. This report shows that the pulmonary microvascular endothelium contains different subpopulations of ECs, many of which are EPCs that can be activated to form capillary-like tubes *in vitro *and *in vivo*. They called them resident microvascular endothelial progenitor cells. Because of the similarity of the early growth characteristics and the identical organ used for isolation, we assume that we have isolated the corresponding progenitor cells from mice. Lung side population (SP) cells are also resident lung precursor cells with both epithelial and mesenchymal potential. Neonatal lung SP and subsets of lung SP from neonatal mice are VEGFR-2/FLK-1^+^, bind EC specific lectins and form networks in matrigels and therefore demonstrate endothelial potential [24]. Earlier gene expression profiling studies of lung SP cells from prenatal mice divided into CD45^+ ^and CD45^- ^cells revealed overexpression of endothelial genes within the CD45^- ^side population [25].

### Mouse lung EPCs possess hemangiogenic and lymphangiogenic potential

Mouse lung EPCs as described in our study are vasculogenic in Matrigel assays *in vitro *and *in vivo*. To date, mouse and rat lungs represent the richest sources of EPCs, which possess high hemangiogenic and lymphangiogenic capacities [11]. Immunophenotyping has indicated that the cell isolates are enriched with EPCs positive for progenitor cell markers. However, the expression of markers specific for blood and lymph endothelial cells indicates heterogeneity of the isolates. Whether this heterogeneity develops during cell culture or exists already during the early phase of cell isolation was a major question to be answered. The limiting dilution assays clearly show that progenitor cells are bipotent and differentiate into blood and lymphatic endothelial cells *in vitro *and form corresponding vessels *de novo *in normal mice.

Together with the recent report from Alvarez et al. our findings supports the premise that the pulmonary microvasculature is a rich endothelial progenitor niche, which may regenerate blood and lymphatic endothelium to maintain vascular homeostasis and contribute to tissue remodelling and repair [11].

A further interesting aspect, which however, could not be resolved in this study, is the exact localisation of the EPC niche within the lung. In addition to a putative direct position in the endothelial monolayer, a localisation in the vessel wall or in perivascular tissue may be possible. Moreover, reliable markers to prospectively identify EPCs in the intact lung are still lacking. The existence of human EPCs and stem cells that are capable of differentiation into mature endothelial cells, hematopoietic and local immune cells has been studied in fragments of the internal thoracic artery of adults [22]. The niche was identified between smooth muscle and adventitial layers of the artery wall. These cells were named vascular wall resident endothelial progenitor cells (VW-EPC). Although CD45^+ ^cells have been found in the same niche, it has been postulated that these cells differentiate into macrophages and hematopoietic cells, but do not participate in capillary sprout formation [22], which needs to be further investigated.

Angiogenic processes within the postnatal pulmonary circulation are difficult to study and the phenomenon is not generally accepted. Angiogenesis may occur within the lung systemic circulation to repair pulmonary circulation defects and to restore blood flow [11]. Lung tissue is densely supplied with blood and lymph vessels and both vessel types possess high plasticity to form new vessels, for example, during inflammation after pathogen infection [26]. Based on this observation, we assumed that genuine precursor cells have the intrinsic capacity to differentiate into blood endothelial cells (BECs) and lymphatic endothelial cells (LECs), which could be demonstrated in this study. Vessel formation has been studied in mouse models for acute or chronic inflammation of the lung. Bacterial infection of the respiratory tract is associated with dramatic morphological changes in the wall of the airways and in the vasculature. Remodelling of both blood vessels and lymphatics can be observed when mice are infected with the bacterium *Mycoplasma pulmonis *[26]. Bacterial infection induces rapid hemangiogenesis and lymphangiogenesis by the increased expression of vessel specific growth factors released either by macrophages or other immune cells and epithelial cells [27]. Inflammation-induced lymphangiogenesis and hemangiogenesis in the lung obviously prevent oedema formation and facilitate the transport and traffic of immune cells to combat acute inflammation, and support respiration [26].

## Conclusion

With our results presented here, we finally demonstrate, that mouse lung EPCs are intrinsically capable of forming blood and lymph vessels *de novo*. It will be important to assess how these cells contribute to normal endothelial cell turnover and respond to injury. Novel mouse models are needed to elucidate challenging questions concerning the direct involvement of lung-derived EPCs in the neoformation of blood and lymph vessels and their fate during remodelling, repair and inflammation.

## Methods

### Immortalized Endothelial Cells and Tumor Cells

The generation of endothelioma cell lines (polyoma middle T-immortalized mouse endothelial cells) from mouse embryos (Balb/c and C57Bl/6) has been described previously [17]. As a model for mouse tumor cells we used pancreatic beta cell tumor (βTC) [28].

### Animals

All studies involving the use of mice were approved by our local institutional animal care committee. The protocols were also approved by the regional council on animal care and correspond to the requirements of the American Physiology Society.

### Isolation and culture of mouse lung microvascular endothelial cells

Mouse lung microvascular endothelial cells (MLMVECs) were isolated from mouse lungs using a magnetic cell separation method and cultured as previously described [29]. Briefly, after euthanasia, three mice were subjected to thoracotomy and the lungs were rinsed with PBS via the right cardiac ventricle and excised. After removal of the larger blood vessels the lungs were minced under sterile conditions with scissors and placed in a 60-mm dish containing DMEM. Tissue was digested with collagenase A (Cat.no. 10103586001; Roche, Germany), rinsed with DMEM and the collected cells were strained through a 40 μm cell strainer (BD Bioscience, Germany). Cells were incubated with anti-CD31-coated Dynabeads (rat, clone MEC13.3 BD; Cat.no.110.035, Dynal, Norway) and separated in a magnetic field. After the washing away of the unbound cells, the beads were released by trypsin/EDTA treatment or cells were seeded with the microbeads. Cell-culture medium was replaced with DMEM enriched with 20% FBS (Cat. no. S1810; Biowest, France). After 8 -10 days, cells were separated again by FACS sorting (FACS Aria, BD Bioscience, Germany) using the same CD31 antibody as before. Immunophenotyping of CD31^+ ^cells was carried out by flow cytometry after cell expansion (passage 4-6) using a previously described multipanel of cell surface and cytoslic markers [17]. Additionally, cells were characterized for markers more specific for blood or lymph endothelial cells [30]. Passages 6 -20 were used for consecutive experiments.

### Cell growth

MLMVEC cells were seeded at 1 × 10^4 ^cells/cm^2 ^in gelatinised separate six-well plates or later in small cell culture flasks (25 cm^2^). The medium contained DMEM and 15-20% FCS (MBE medium) and has been described previously [17]. Cells were fed at least twice a week and split 1:4 up to 1:16 after confluence. Growth studies were performed in duplicate or triplicate and for studies with growth factors, endothelial cell growth supplement (ECGF, Cat. no. 300-090; Reliatech, Germany) was omitted from the media (ECGF-free cell growth protocol). Growth was determined by electronic cell counting from day 0, at 48-h intervals. Results show the average of duplicate/triplicate measurements repeated at least twice.

### Proliferation assays

MLMVECs and endothelioma cells were seeded at 1.5 × 10^4^/well in separate 48 well plates (1,1 cm^2^/well) in MBE medium. Following 72 h incubation at 37°C with 5% CO_2_, the medium was replaced and this time point was designated day 3. Proliferation studies were performed in duplicate. Cell growth was determined after day 3 at 24 h intervals, using an electronic cell counter (Casy Systems, Germany). Results denote the average of duplicate measurements repeated at least once. For the proliferation assay in the presence of growth factors, cells were seeded in minimal medium (in 5% FCS w/o ECGF) supplemented with the growth factors and measured over 4-6 days with one growth factor exchange after three days. For proliferation assays in the presence of VEGF-A (mouse VEGF_164_) we used 5 and 20 ng/ml growth factor.

### Limiting dilution analysis

Limiting dilution analysis was performed as described earlier with some modifications [31]. The single-cell clonogenic assay was performed with MLMVECs from one isolation at 80-100% confluence. Briefly, cells were trypsinized, and from the suspension the cell number was electronically determined (Casy 1 Cell Counter). We diluted our cells to one cell per 100 μl of standard culture medium and then dispended 100 μl/well into two and a half gelatine-coated 96-well plates. Cells were examined daily under the microscope to study their colony-forming ability. After one week, wells were supplemented with another 100 μl of standard culture medium. Cells were further cultured at 37°C with 5% CO_2 _and 21% O_2 _for another three weeks. After 4 weeks all wells, which contained at least one cell, were counted. Quantification of moderately and highly proliferating colonies was performed independently by two persons by visual inspection at x40 magnification. Results denoted the average from 240 wells. Highly proliferating, clonogenic MLMVECs were obtained after further expanding colonies from confluent wells.

### Immunophenotyping by FACS Analysis

MLMVECs and other mouse endothelial cells were treated with accutase (Cat.no. L11-007, PAA, Austria) and suspended at 1 × 10^5 ^cells per tube in 1 ml microtubes containing 200 μl of cold PBS (calcium and magnesium free) supplemented with 2% FCS. For the immunostaining, cells were incubated for 15 min on ice. All cells were rinsed with cold PBS/2%FCS and incubated with the primary antibody or with fluorescein-conjugated lectin or isotype control antibody for 30 min on ice in the dark. Cells were rinsed in cold PBS/2% FCS and then incubated with the corresponding fluorescent dye-conjugated secondary antibody. At the end of antibody incubation, cells were transferred to flow cytometric tubes (Micronic, The Netherlands) and subjected to flow analysis using FACS Calibur (Becton Dickinson). We used the following primary antibodies: CD31 (rat clone MEC13.3; BD), CD34 (rat clone RAM34; BD), CD45-FITC (rat clone 104, BD Pharmingen), CD102 (rat clone 3C4(mlC2/4); BD), CD105 (rat #6J9; Reliatech), CD144 (rat clone 11D4.1; BD), VEGFR-2 (rat clone Avas12α1; BD), VEGFR-3 (rat Cat. no. 103-M36, Reliatech), Lyve1 (rabbit Cat. no. 103-PA50AG; Reliatech), podoplanin (hamster Cat. no. 103-M40; Reliatech), PV1/Meca32 (rat; U. Deutsch), LA102 (rat Cat.no. 103-M152; Reliatech) and LA5 (rat, Cat.no. 103-M154; Reliatech). The following primary antibodies for HSC were used: CD117-PE (rat 120-002-406; Miltenyi Biotech), CD133 (rat clone 13A4; eBioscience, U.S. and rat clone MB9-3Ga, Miltenyi Biotech, Germany) and CD41-PE (rat clone MWReg30; eBioscience, US). For lectin binding we used the FITC-conjugated *Griffonia simpliciflolia *(Cat. no. FL-1101; Biozol, Germany). For immunodetection of the von Willebrand factor, cells were incubated with the primary and secondary antibodies in the presence of 0.15% TritonX-100. We used the following conjugated secondary antibodies: goat anti rat-FITC (Jackson IR, U.S.), goat-anti rabbit-FITC (Jackson IR), goat-anti-hamster-PE (Jackson IR). As a control, cells were incubated with secondary antibodies exclusively (negative control). Confirmation of protein expression or lectin interaction in MLMVECs was the right-shift of the curve as compared with that of a corresponding control.

### Measurement of NO production

The cell-permeable diacetate derivate, DAF-2 DA (4,5-Diaminofluorescein.diacetate) was used to measure NO production (Cat. no. ALX-620-056; Alexis, Switzerland). After loading and subsequent hydrolysis by cytosolic esterases, DAF-2 is released, which is relatively non-fluorescent at physiological pH. In the presence of NO and O_2_, DAF-2 is converted to the fluorescent triazole derivated, DAF-2T [32]. Briefly, EPCs grown in 6-well plates overnight were than incubated with 5 μM fresh DAF-DA in 1 ml PBS for 30 min. After washing, cells were detached and transferred to FACS buffer for FCS analysis (see above). NIH/3T3 cells have been initially used as control cells and have been found to be negative for NO.

### Matrigel *in vitro *outgrowth assay

To study sprouting angiogenesis *in vitro*, mouse endothelium spheroids of a defined cell number were generated as described previously [33]. Briefly, ECs were suspended in MBE culture medium containing 0.25% (wt/vol) methyl cellulose and cultured overnight in hanging drops of 50 μl. The cells formed single spheroids with a defined cell number. Matrigel (BMM Matrigel; BD Bioscience, Germany) was diluted 1:3 with serum-free endothelial cell medium (Cat. no. U15-002, PAA, Austria), supplemented with various growth factors (HGF, ECGF, VEGF-A, bFGF, VEGF-C, TNF-α) and 150 μl was mixed with each spheroid and seeded in 48-well plates (as duplicates). Wells were analysed for sprout formation using phase-contrast microscopy at 1, 5 and 14 days after seeding. The average length of sprouts was measured with a digitalized Zeiss photomicroscope and compared to the negative controls. Results denote the average of multiple measurements of at least two separate experiments.

### Green fluorescent protein and luciferase transfection

Lentiviral constructs encoding green fluorescent protein (GFP; pPREGFPSIN) and luciferase (LUC; pJSCMVLuc) without antibiotica resistance, were produced as previously described [34]. Low passage MLMVECs and high passage MLMVECs (> 20) were seeded in a 12-well plate, supplemented with standard culture medium and incubated to about 60-70% confluence. The medium was removed and culture supernatant containing lentiviral particles constructs, was added to the cells in the presence of 8 μg/ml Polybrene for 8 h (Sigma). The infected cells were cultivated as a pool of infected cells. The infection efficiency varied between 40-60% depending on the expression cassette and the cell type. For maintenance the medium was changed twice a week until cells were confluent and used for liquid nitrogen freezing and for *in vivo *Matrigel injections.

### Matrigel-Mediated *de novo *Vessel Formation *in vivo*

MLMVECs were trypsinized, counted and cultured overnight in hanging drops to allow predifferentiation of cells in spheroids (~ 10,000 cells/spheroid). Matrigel was supplemented with 500 ng/ml bFGF and VEGF-A and used with or without spheroids. For each injection, about 30-50 spheroids were mixed in 300-400 μl unpolymerized Matrigel at 4°C. The mixed solution was injected subcutaneously (left and right dorsal region, two plugs per animal) into mildly sedated (ketamine 100 mg/kg ip) Balb/c female mice using a 20-gauge needle. The injected Matrigel polymerizes at body temperature and becomes a plug. As controls, Matrigel was injected on the left side without cells but with growth factors. 10-12 days after injection, Matrigel plugs were excised and treated overnight with methanol/10% DMSO. Plugs were further immersion-fixed in 4% paraformaldehyde, treated with saccharose and embedded in frozen section medium (Neg50; Richard-Allan Scientific, U.S.).

### Immunodetection of MLMCECs *in vivo*

Excised Matrigel plugs embedded in frozen section medium were used to prepare cryosections of 16-20 μm thickness. Non-specific binding of antibodies was blocked by incubation with 1% bovine serum albumin (BSA) for 1 h before incubation with primary antibodies. The following primary antibodies were used for single and double-staining: Anti-mouse CD31 (rat clone MEC13.3; BD), anti-mouse CD45 (rat clone 104; BD Pharmingen) anti-mouse podoplanin (hamster Cat. no. 103-M40; Reliatech), anti-mouse CD105 (rat #6J9; Reliatech), anti-GFP (rabbit Cat. no. ab6556-25, Abcam, U.K.) and anti-mouse LA102 (rat Cat. no 103-M152).

The sections were incubated with primary antibodies for 1 h. After rinsing, secondary antibodies were applied at 1:100 or 1:200 dilution for 1 h. We used goat anti-rat TRITC-conjugated, (Jackson IR, U.S.), donkey anti-rabbit Alexa488 (Pierce, Rockford, U.S.), goat anti-hamster Alexa594, goat anti-rabbit Alexa594 and goat anti-rat Alexa350-labeled (MoBiTec, Göttingen, Germany). DAPI reagent (Hoechst Dye) was added to stain nuclei.

After being rinsed, the sections were mounted under coverslips with Fluoromount-G (Southern Biotechnology, U.S.). They were studied with a DM500 B epifluorescent microscope and Axioplan 2 LSM 510 laser-scanning microscope (Zeiss, Germany).

### Anchorage-independent growth on soft agar

MLMVECs and mouse tumour cells (βTC) as positive control were suspended in MBE medium and seeded at a density of ~1 × 10^3 ^cells/cm^2 ^in 48 well plates coated with 0.8% soft agar in MBE medium. Cells were incubated at 37°C and 5% CO_2_. After 8-10 days, plates were studied with phase-contrast microscopy. Efficiency of anchorage-independent growth was determined by the capacity of cells to form colonies (> 100 cells) within the agar. Experiments were performed as duplicates and repeated at least once.

## Abbreviations

BEC: blood endothelial cell; LEC: lymph endothelial cell; FACS: fluorescence activated cell sorting; EPC: endothelial progenitor cell; MLMVEC: mouse lung microvascular endothelial cell; VEGFR-2/-3: vascular endothelial growth factor receptor-2/-3; ECGF: endothelial cell growth supplement from bovine brain; HGF: hepatocyte growth factor; VEGF-A: vascular endothelial growth factor-A; bFGF: basic fibroblast growth factor; TNF-α: tumor necrosis factor alpha; GFP: green fluorescence protein; PmT: polyoma middle T oncogene.

## Authors' contributions

JS contributed to the studies involving isolation of EPCs, flow cytometry studies and immunofluorscence. MR contributed to in vivo studies and immunofluorscence. KB made substantial contributions to immunofluoescence studies and analysis of data. GR contributed to in vitro differentiation studies and acquisition of data. AMS and SN performed the research, and contributed to the acquisition and interpretation of data. MB contributed to the animal studies and interpretation of data. BB provided specific research material and was involved in interpretation of data. TM contributed to the studies involving lentiviral transduction of primary mouse cells. JW and HAW contribute to the overall conception and design of the experiments, in drafting the manuscript and give final approval of the version to be published. All authors have read and approved the final manuscript.

## Supplementary Material

Additional file 1 **Heterogeneity and pattern formation of mouse EPCs after isolation and CD31 cell sorting shown at lower magnification**. a) Pattern formation of almost confluent cells after the first CD31 selection shown by phase contrast microscopy. b) The same cells as in a). Heterogeneity of cells is demonstrated by immunofluorescence several days after Lyve1-positive selection. Note CD31-positive BECs surrounding Lyve1-positive LECs, which express variable amounts of CD31 (yellow). 100-fold. Click here for file

Additional file 2 **Isolation of MLMVECs from mouse lungs by FACS sorting**. Low passage (passage 3) MLMVECs were sorted by FACS into CD31^+^/Lyve1^+ ^(gate R1) and CD31^+^/Lyve1^- ^(gate R2) cells for further subculturing and immunophenotyping. The percentage of cells in the different regions is indicated (%). Click here for file

Additional file 3 **Subculturing of Lyve1^+ ^and Lyve1^- ^cells after CD31/Lyve1 cell sorting**. Early passage MLMVEC (passage 3) were FACS sorted (same cells as in supplementary figure 2) and separately cultured. Ten days after sub-culturing (passage 4) cells were studied again by FACS analysis with anti-Lyve1 antibodies. The CD31^+^/Lyve1^+ ^cells indicated a stable high Lyve1 expression (a) whereas CD31^+^/Lyve^- ^cells now expressed Lyve1 at a comparably high rate (c). One passage later (passage 5) some CD31^+^/Lyve^+ ^cells were negative for Lyve1 (b) whereas CD31^+^/Lyve1^- ^cells were getting even more positive for Lyve1 (d). Click here for file

Additional file 4 **Sprout formation of MLMVEC spheroids on Matrigel in the presence of different growth factor combinations**. Quantitative sprout formation of MLMVECs (in μm) in the presence of different growth factors 5 days after plating together with Matrigel. Growth factor concentrations in Matrigel: VEGF-C (500 ng/ml); VEGF-A (50 ng/ml); bFGF (20 ng/ml); TNF-α (10 ng/ml); HGF (20 ng/ml); ECGF= endothelial cell growth factor supplement (50 μg/ml + heparin). Sprouts were measured with an inverted stereo microscope (Zeiss) using specific software (AxioVision Rel. 4.5) for the determination of sprout lengths. Cells from A were isolated from C57Bl/6 mice and cells from B were isolated from Balb/c mice. Values= means ± SD. Click here for file

Additional file 5 **Double labelling of lung EPCs with Lyve1, MECA32 and LA5**. Immunophenotyping of cells was performed with cytometric analysis. Mouse lung EPCs are double positive for Lyve1 and MECA32. Only one part of the Lyve1^+ ^cells are also positive for LA5. Cells from two isolations have an almost an identical pattern of the three markers. Click here for file

Additional file 6 **Mouse lung EPCs have the capacity to produce NO in vitro**. Fluorescence detection of NO production was done with a practical FACS assay with living cells. Membrane-permeable DAF-2 diacetate has been used for indirect detection of NO production. Click here for file

Additional file 7 **Extended *in vivo *studies of mouse lung EPCs after lentiviral transduction with GFP marker gene**. *De novo *formation of lymphatic vessels as revealed by double staining with anti-GFP and anti-LA102 (which is LEC-specific) in combination with nuclear Dapi staining. (Upper 4 figures). Note that staining with anti-CD45 (leukocyte common antigen) resulted in the staining of some scattered cells in the interstitium but no double staining with LA102 could be observed. (Lower 4 figures) 200-fold. Click here for file

Additional file 8 **In vivo studies of mouse lung EPCs to detect blood and lymphatic networks in the implanted Matrigel**. Formation of blood and lymphatic networks in the same Matrigel by triple staining with anti-GFP, anti-podoplanin (LEC-specific) and anti-CD31. Note that the merged figure contained GFP-tube forming cells which are CD31 and podoplanin positive (LEC) and podoplanin negative cells (BEC). 200-fold. Click here for file
