# Supplementary material for: Mouse lung contains endothelial progenitors with high capacity to form blood and lymphatic vessels
Source: BMC Cell Biol. 2010 Jul 1;11:50. doi: 10.1186/1471-2121-11-50 (PMC2911414; doi:10.1186/1471-2121-11-50)
Supplement: Additional file 1 — Heterogeneity and pattern formation of mouse EPCs after isolation and CD31 cell sorting shown at lower magnification. a) Pattern formation of almost confluent cells after the first CD31 selection shown by phase contrast microscopy. b) The same cells as in a). Heterogeneity of cells is demonstrated by immunofluorescence several days after Lyve1-positive selection. Note CD31-positive BECs surrounding Lyve1-positive LECs, which express variable amounts of CD31 (yellow). 100-fold. [file 1471-2121-11-50-S1.PDF]

Additional file 1

Heterogeneity and pattern formation of mouse EPCs after isolation and CD31 cell sorting shown at lower magnification

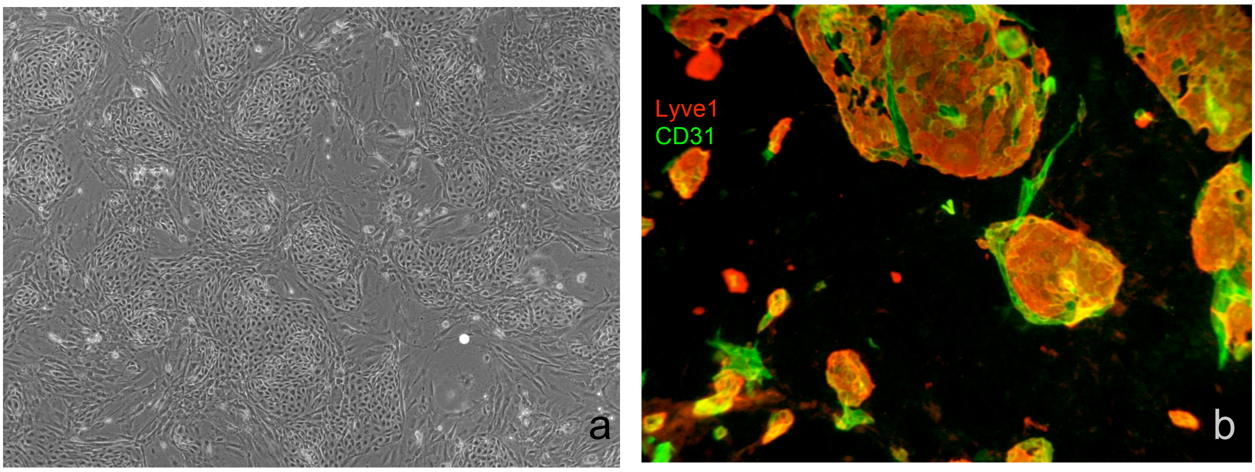

a) Pattern formation of almost confluent cells after the first CD31 selection shown by phase contrast microscopy. b) The same cells as in a). Heterogeneity of cells is demonstrated by immunofluorescence several days after Lyve1-positive selection. Note CD31-positive BECs surrounding Lyve1-positive LECs, which express variable amounts of CD31 (yellow). 100-fold
