# Supplementary material for: Mouse lung contains endothelial progenitors with high capacity to form blood and lymphatic vessels
Source: BMC Cell Biol. 2010 Jul 1;11:50. doi: 10.1186/1471-2121-11-50 (PMC2911414; doi:10.1186/1471-2121-11-50)
Supplement: Additional file 2 — Isolation of MLMVECs from mouse lungs by FACS sorting. Low passage (passage 3) MLMVECs were sorted by FACS into CD31+/Lyve1+ (gate R1) and CD31+/Lyve1- (gate R2) cells for further subculturing and immunophenotyping. The percentage of cells in the different regions is indicated (%). [file 1471-2121-11-50-S2.PDF]

Additional file 2

Isolation of MLMVECs from mouse lungs by FACS sorting

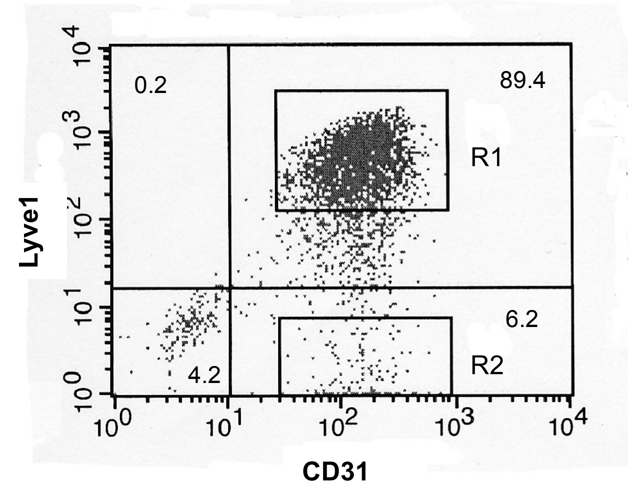

Low passage (passage 3) MLMVECs were sorted by FACS into $CD31^+/Lyve1^+$ (gate R1) and $CD31^+/Lyve1^-$ (gate R2) cells for further subculturing and immunophenotyping. The percentage of cells in the different regions is indicated (%).
