# Supplementary material for: Mouse lung contains endothelial progenitors with high capacity to form blood and lymphatic vessels
Source: BMC Cell Biol. 2010 Jul 1;11:50. doi: 10.1186/1471-2121-11-50 (PMC2911414; doi:10.1186/1471-2121-11-50)
Supplement: Additional file 3 — Subculturing of Lyve1+ and Lyve1- cells after CD31/Lyve1 cell sorting. Early passage MLMVEC (passage 3) were FACS sorted (same cells as in supplementary figure 2) and separately cultured. Ten days after sub-culturing (passage 4) cells were studied again by FACS analysis with anti-Lyve1 antibodies. The CD31+/Lyve1+ cells indicated a stable high Lyve1 expression (a) whereas CD31+/Lyve- cells now expressed Lyve1 at a comparably high rate (c). One passage later (passage 5) some CD31+/Lyve+ cells were negative for Lyve1 (b) whereas CD31+/Lyve1- cells were getting even more positive for Lyve1 (d). [file 1471-2121-11-50-S3.PDF]

Additional file 3

Subculturing of Lyve1$^+$ and Lyve1$^-$ cells after CD31/Lyve1 cell sorting

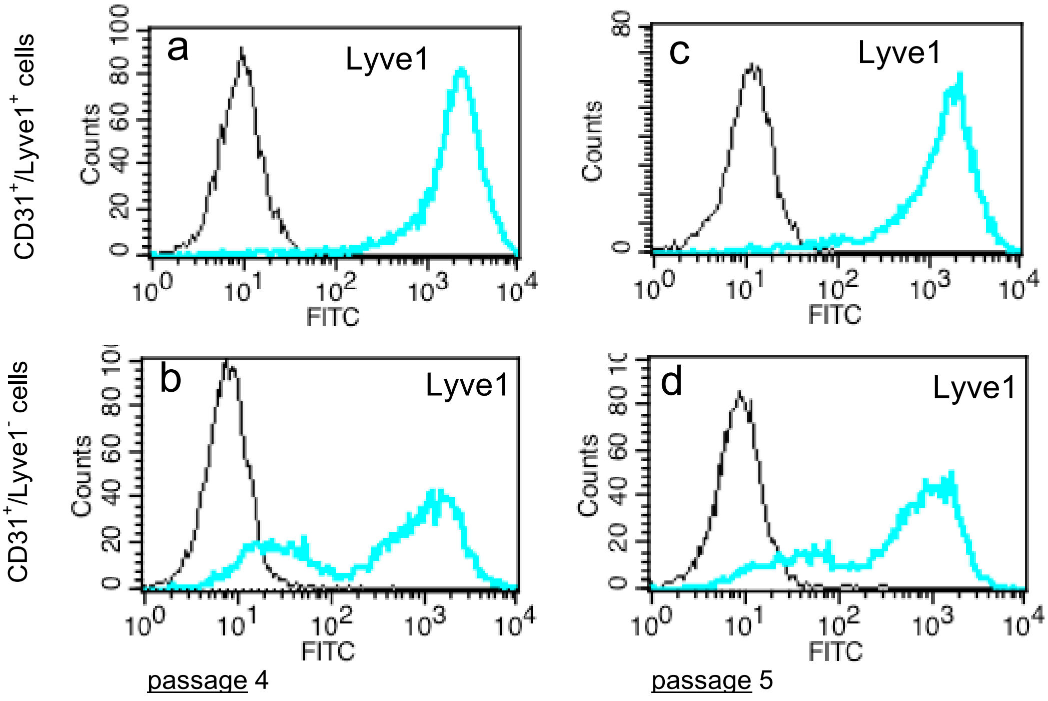

Early passage MLMVEC (passage 3) were FACS sorted (same cells as in supplementary figure 2) and separately cultured. Ten days after sub-culturing (passage 4) cells were studied again by FACS analysis with anti-Lyve1 antibodies. The CD31$^+$/ Lyve1$^+$ cells indicated a stable high Lyve1 expression (a) whereas CD31$^+$/Lyve$^-$ cells now expressed Lyve1 at a comparably high rate (c). One passage later (passage 5) some CD31$^+$/Lyve$^+$ cells were negative for Lyve1 (b) whereas CD31$^+$/Lyve1$^-$ cells were getting even more positive for Lyve1 (d).
