# Supplementary material for: Mouse lung contains endothelial progenitors with high capacity to form blood and lymphatic vessels
Source: BMC Cell Biol. 2010 Jul 1;11:50. doi: 10.1186/1471-2121-11-50 (PMC2911414; doi:10.1186/1471-2121-11-50)
Supplement: Additional file 4 — Sprout formation of MLMVEC spheroids on Matrigel in the presence of different growth factor combinations. Quantitative sprout formation of MLMVECs (in μm) in the presence of different growth factors 5 days after plating together with Matrigel. Growth factor concentrations in Matrigel: VEGF-C (500 ng/ml); VEGF-A (50 ng/ml); bFGF (20 ng/ml); TNF-α (10 ng/ml); HGF (20 ng/ml); ECGF= endothelial cell growth factor supplement (50 μg/ml + heparin). Sprouts were measured with an inverted stereo microscope (Zeiss) using specific software (AxioVision Rel. 4.5) for the determination of sprout lengths. Cells from A were isolated from C57Bl/6 mice and cells from B were isolated from Balb/c mice. Values= means ± SD. [file 1471-2121-11-50-S4.PDF]

Additional file 4

Sprout formation of MLMVEC spheroids on Matrigel in the presence of different growth factor combinations.

growth factor/combination	MLMVEC (isolat A)	MLMVEC (isolat B)
untreated	121 ± 9	210 ± 12
VEGF-C/bFGF	254 ± 16	211 ± 13
VEGF-A/TNF-α	92 ± 5	97 ± 7
VEGF-A/bFGF	121 ± 7	191 ± 10
TNF-α/bFGF	102 ± 3	152 ± 13
HGF	158 ± 11	364 ± 11
ECGF	182 ± 2	352 ± 10

Quantitative sprout formation of MLMVECs (in μm) in the presence of different growth factors 5 days after plating together with Matrigel. Growth factor concentrations in Matrigel: VEGF-C (500 ng/ml); VEGF-A (50 ng/ml); bFGF (20 ng/ml); TNF-α (10 ng/ml); HGF (20 ng/ml); ECGF= endothelial cell growth factor supplement (50 μg/ml + heparin). Sprouts were measured with an inverted stereo microscope (Zeiss) using specific software (AxioVision Rel. 4.5) for the determination of sprout lengths. Cells from A were isolated from C57Bl/6 mice and cells from B were isolated from Balb/c mice. Values= means ± SD.
