# Supplementary material for: Mouse lung contains endothelial progenitors with high capacity to form blood and lymphatic vessels
Source: BMC Cell Biol. 2010 Jul 1;11:50. doi: 10.1186/1471-2121-11-50 (PMC2911414; doi:10.1186/1471-2121-11-50)
Supplement: Additional file 5 — Double labelling of lung EPCs with Lyve1, MECA32 and LA5. Immunophenotyping of cells was performed with cytometric analysis. Mouse lung EPCs are double positive for Lyve1 and MECA32. Only one part of the Lyve1+ cells are also positive for LA5. Cells from two isolations have an almost an identical pattern of the three markers. [file 1471-2121-11-50-S5.PDF]

Additional file 5

Double labeling of lung EPCs with Lyve1, MECA32 and LA5

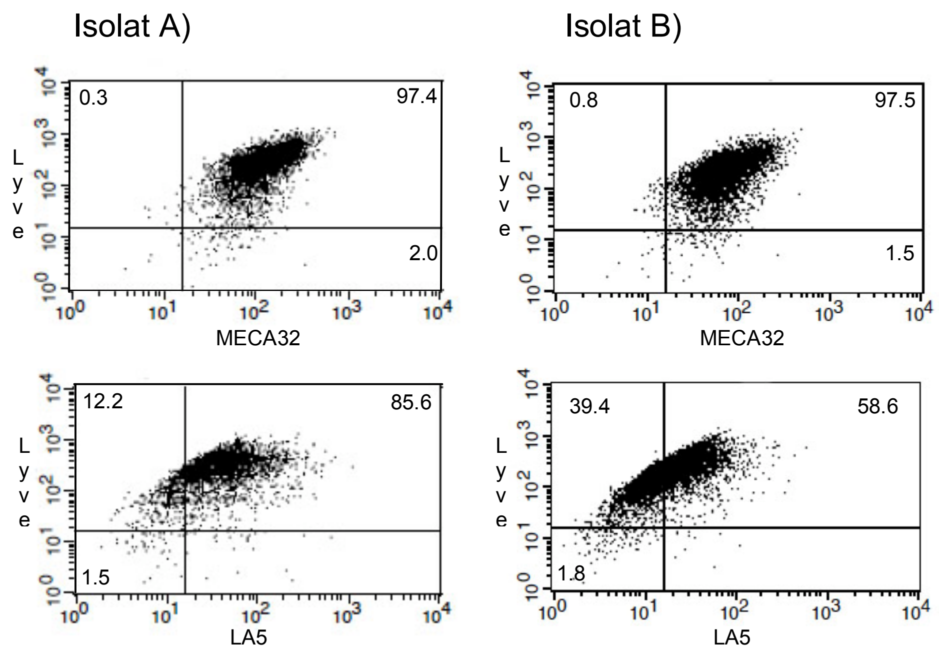

Immunophenotyping of cells was performed with cytometric analysis. Mouse lung EPCs are double positive for Lyve1 and MECA32. Only one part of the Lyve1$^+$ cells are also positive for LA5. Cells from two isolations have an almost an identical pattern of the three markers.
