# Supplementary material for: Mouse lung contains endothelial progenitors with high capacity to form blood and lymphatic vessels
Source: BMC Cell Biol. 2010 Jul 1;11:50. doi: 10.1186/1471-2121-11-50 (PMC2911414; doi:10.1186/1471-2121-11-50)
Supplement: Additional file 6 — Mouse lung EPCs have the capacity to produce NO in vitro. Fluorescence detection of NO production was done with a practical FACS assay with living cells. Membrane-permeable DAF-2 diacetate has been used for indirect detection of NO production. [file 1471-2121-11-50-S6.PDF]

Additional file 6

Mouse lung EPCs have the capacity to produce NO in vitro

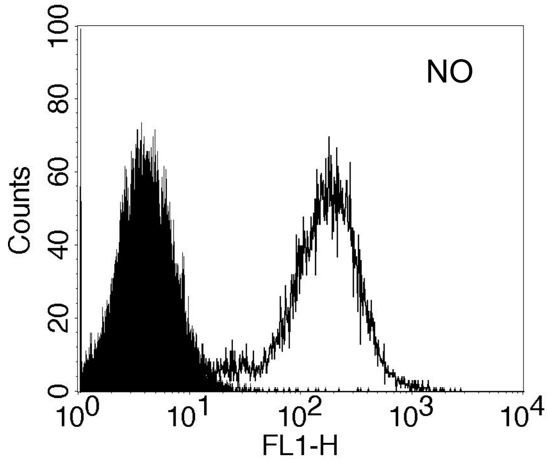

Fluorescence detection of NO production was done with a practical FACS assay with living cells. Membrane-permeable DAF-2 diacetate has been used for indirect detection of NO production.
