# Supplementary material for: Mouse lung contains endothelial progenitors with high capacity to form blood and lymphatic vessels
Source: BMC Cell Biol. 2010 Jul 1;11:50. doi: 10.1186/1471-2121-11-50 (PMC2911414; doi:10.1186/1471-2121-11-50)
Supplement: Additional file 7 — Extended in vivo studies of mouse lung EPCs after lentiviral transduction with GFP marker gene. De novo formation of lymphatic vessels as revealed by double staining with anti-GFP and anti-LA102 (which is LEC-specific) in combination with nuclear Dapi staining. (Upper 4 figures). Note that staining with anti-CD45 (leukocyte common antigen) resulted in the staining of some scattered cells in the interstitium but no double staining with LA102 could be observed. (Lower 4 figures) 200-fold. [file 1471-2121-11-50-S7.PDF]

Additional file 7

Extended *in vivo* studies of mouse lung EPCs after lentiviral transduction with GFP marker gene.

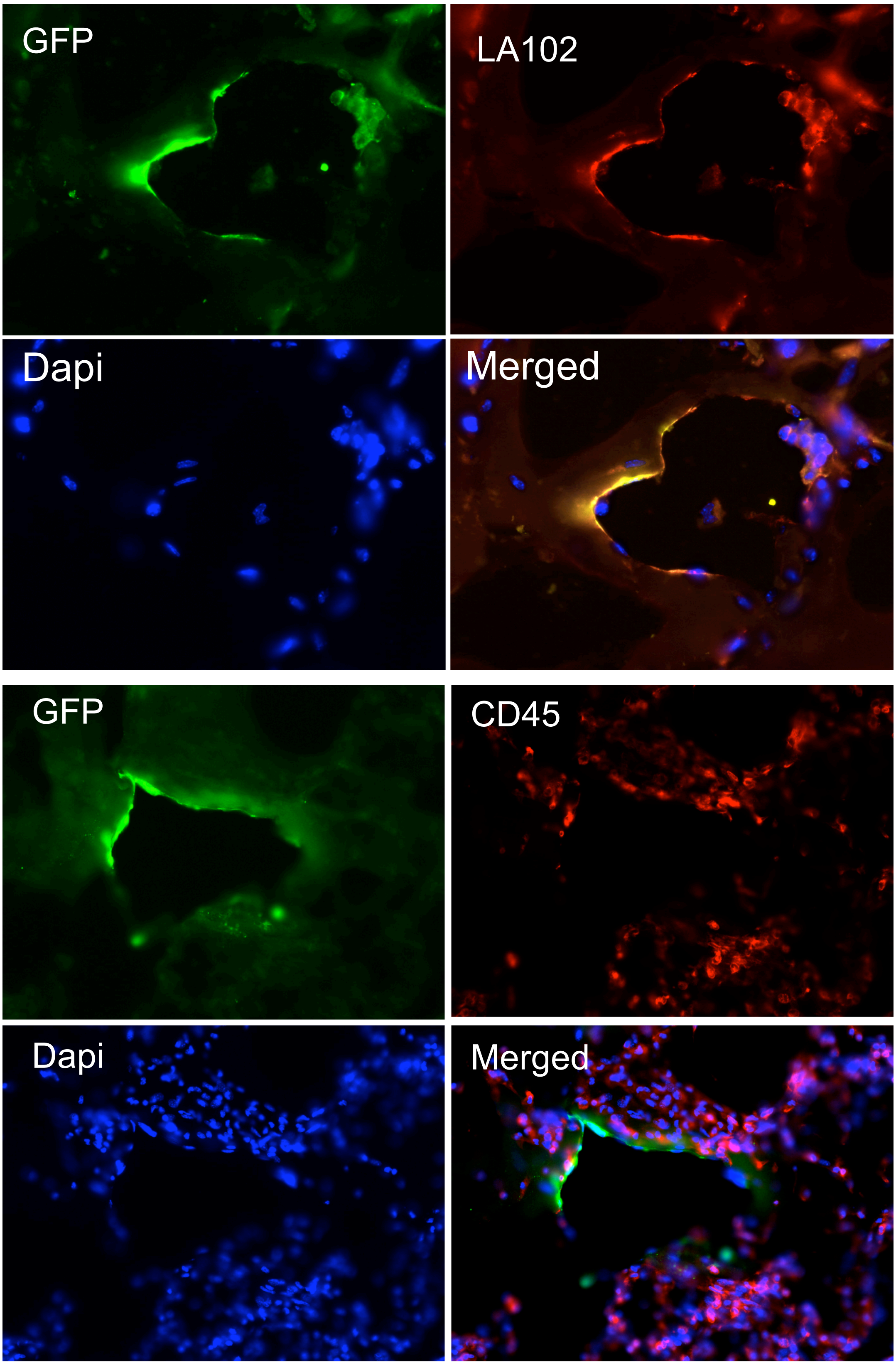

De novo formation of lymphatic vessels as revealed by double staining with anti-GFP and anti-LA102 (which is LEC-specific) in combination with nuclear Dapi staining. (Upper 4 figures). Note that staining with anti-CD45 (leukocyte common antigen) resulted in the staining of some scattered cells in the interstitium but no double staining with LA102 could be observed. (Lower 4 figures) 200-fold
