# Supplementary material for: Mouse lung contains endothelial progenitors with high capacity to form blood and lymphatic vessels
Source: BMC Cell Biol. 2010 Jul 1;11:50. doi: 10.1186/1471-2121-11-50 (PMC2911414; doi:10.1186/1471-2121-11-50)
Supplement: Additional file 8 — In vivo studies of mouse lung EPCs to detect blood and lymphatic networks in the implanted Matrigel. Formation of blood and lymphatic networks in the same Matrigel by triple staining with anti-GFP, anti-podoplanin (LEC-specific) and anti-CD31. Note that the merged figure contained GFP-tube forming cells which are CD31 and podoplanin positive (LEC) and podoplanin negative cells (BEC). 200-fold. [file 1471-2121-11-50-S8.PDF]

Additional file 8

In vivo studies of mouse lung EPCs to detect blood and lymphatic networks in the implanted Matrigel

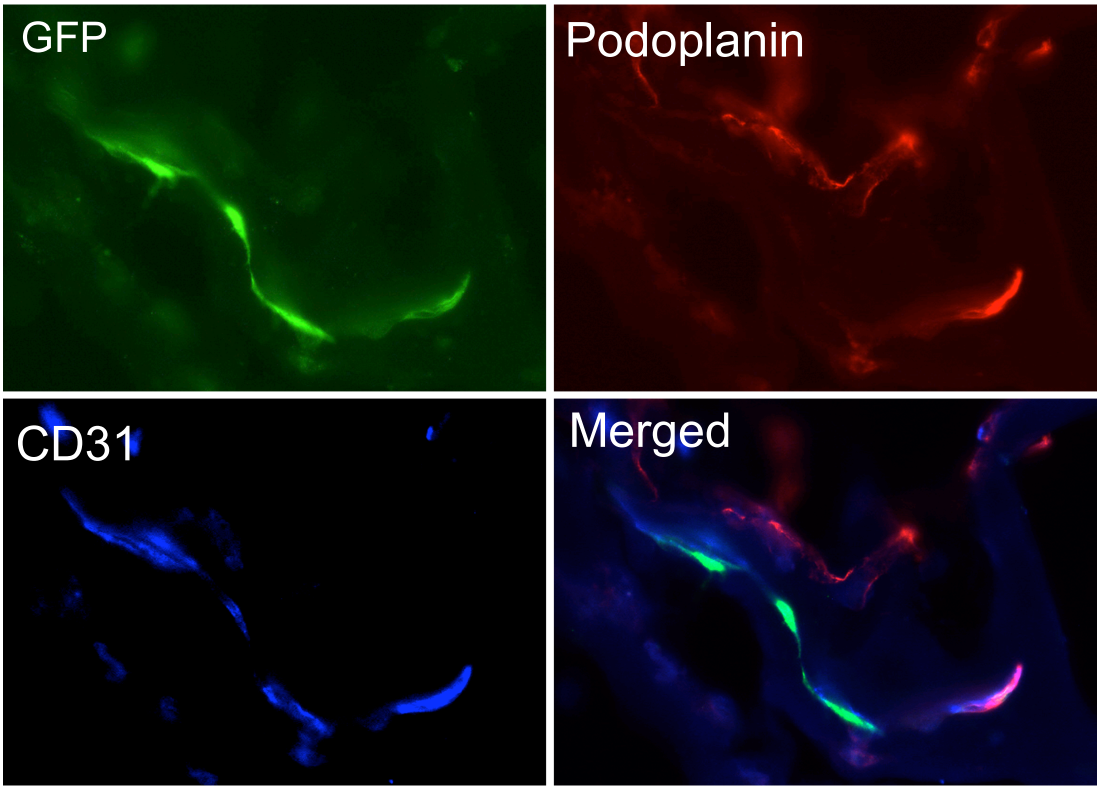

Formation of blood and lymphatic networks in the same Matrigel by triple staining with anti-GFP, anti-podoplanin (LEC-specific) and anti-CD31. Note that the merged figure contained GFP-tube forming cells which are CD31 and podoplanin positive (LEC) and podoplanin negative cells (BEC). 200-fold.
